# Porcine Reproductive and Respiratory Syndrome Virus nsp1β Stabilizes HIF-1α to Enhance Viral Replication

**DOI:** 10.1128/spectrum.03173-22

**Published:** 2022-11-23

**Authors:** Yu Pang, Yanrong Zhou, Yuchen Wang, Zheng Sun, Jiao Liu, Chenyu Li, Shaobo Xiao, Liurong Fang

**Affiliations:** a State Key Laboratory of Agricultural Microbiology, College of Veterinary Medicine, Huazhong Agricultural University, Wuhan, China; b The Key Laboratory of Preventive Veterinary Medicine in Hubei Province, Cooperative Innovation Center for Sustainable Pig Production, Wuhan, China; Changchun Veterinary Research Institute

**Keywords:** porcine reproductive and respiratory syndrome virus, glycolysis, hypoxia-inducible factor 1α, von Hippel-Lindau tumor suppressor, deubiquitination, nonstructural protein 1β

## Abstract

Porcine reproductive and respiratory syndrome virus (PRRSV) is an *Arterivirus* that has been devastating the swine industry worldwide since the late 1980s. Severe interstitial pneumonia is the typical pathological characteristic of PRRSV-infected swine. Accumulating evidence has suggested that hypoxia-inducible factor 1α (HIF-1α) plays vital roles in the development of inflammation and the viral life cycle. However, the role and the underlying mechanism of HIF-1α in PRRSV infection remain elusive. Here, we found that PRRSV infection elevated HIF-1α expression. Furthermore, overexpression of HIF-1α increased PRRSV replication, whereas knockdown of HIF-1α inhibited PRRSV infection. Our further mechanistic analysis revealed that PRRSV-encoded nonstructural protein 1β (nsp1β) promoted HIF-1α transcription via its N-terminal nuclease activity and degraded the polyubiquitin chain of HIF-1α via its C-terminal deubiquitylation (DUB) enzyme activity, collectively stabilizing HIF-1α. Meanwhile, nsp1β interacted with both HIF-1α and von Hippel-Lindau tumor suppressor (pVHL) to form a ternary complex, which may have hindered pVHL-mediated ubiquitination degradation of HIF-1α by impairing the interaction between HIF-1α and pVHL. Interestingly, pVHL also stabilized nsp1β via K63-linked ubiquitination, forming a positive feedback loop to stabilize HIF-1α. Taken together, these results indicate that PRRSV infection stabilizes HIF-1α to facilitate viral proliferation and that viral nsp1β plays a vital role in enhancing the expression and stabilization of HIF-1α. The regulation of HIF-1α may have great therapeutic potential for the development of novel drugs against PRRSV.

**IMPORTANCE** Porcine reproductive and respiratory syndrome virus (PRRSV) has devastated the swine industry worldwide for over 30 years and shows no signs of slowing down. In this study, we found that PRRSV infection elevated hypoxia-inducible factor 1α (HIF-1α) expression. In addition, overexpressed HIF-1α contributed to PRRSV replication, whereas knockdown of HIF-1α reduced PRRSV growth. The PRRSV-encoded nonstructural protein 1β (nsp1β) exerted a stabilizing effect on HIF-1α through its nuclease protease and papain-like cysteine protease enzymatic domains. PRRSV nsp1β also interacted with von Hippel-Lindau tumor suppressor (pVHL) and HIF-1α, whereby nsp1β impaired the interaction between HIF-1α and pVHL. This work deepens our understanding of the molecular mechanisms involved in PRRSV infection and provides new insights for the development of HIF-1α-based anti-PRRSV therapies.

## INTRODUCTION

Porcine reproductive and respiratory syndrome virus (PRRSV), a single-stranded positive-strand RNA virus belonging to the *Arteriviridae* family, causes respiratory disease in piglets and adult swine and late abortions and stillbirths in sows (1, 2). As a very successful virus, PRRSV has devastated the swine industry worldwide over 30 years and has shown no sign of slowing down (3, 4). Although many commercial vaccines against PRRSV have been made available, unfortunately, neither traditional control strategies nor conventional vaccines provide sustainable control of PRRSV (5–7). Thus, an understanding of virus-host interactions may provide new potential avenues for the treatment of PRRSV infection.

PRRSV infection causes the excessive production of proinflammatory cytokines known as a “cytokine storm” (8). Several studies consistently showed remarkably increased abundances of interleukin-1β (IL-1β), IL-8, IL-6, and RANTES during PRRSV infection (9, 10). The accumulation of large amounts of proinflammatory cytokines induced by PRRSV in the lung results in the clinical sign of severe respiratory distress and the pathological changes of acute lung injury (interstitial pneumonia) (11, 12). Thus, there appeared to be a correlation between host mortality and the expression of proinflammatory cytokines. Meanwhile, the mechanism by which PRRSV induces a “cytokine storm” is unclear.

Hypoxia-inducible factor 1 (HIF-1) is a major transcriptional activator, allowing cells to adapt to hypoxia (13). HIF-1 is a heterodimeric protein that consists of two proteins, HIF-1α and HIF-1β. HIF-1β is the constitutively expressed subunit, but the expression of HIF-1α is stabilized rapidly in response to hypoxia or infection (14). Under normoxic conditions, HIF-1α is hydroxylated by three prolyl hydroxylase domain (PHD) enzymes and then recognized by the von Hippel-Lindau tumor suppressor (pVHL)-containing E3 ligase, which leads to its polyubiquitination and degradation (13). Under hypoxic conditions, inhibition of PHD activity diminishes the hydroxylation of HIF-1α, stabilizing it to form a heterodimer with HIF-1β, which activates transcription via binding to hypoxia-responsive elements in numerous target genes, including glucose transporter 1 (*GLUT-1*), vascular endothelial growth factor A (*VEGFA*), and a disintegrin and metalloprotease (ADAM) family members *ADAM10* and *ADAM17* (14–18). Previous studies showed that some viruses are able to stabilize the expression of HIF-1α, causing different effects, including promoting viral replication and supporting the expression/maturation/release of proinflammatory cytokines (19–21). For example, severe acute respiratory syndrome coronavirus 2 (SARS-CoV-2) and human immunodeficiency virus type 1 (HIV-1) infection induce HIF-1α stabilization, and stabilized HIF-1α upholds viral replication; moreover, HIF-1α plays an important role in SARS-CoV-2-induced and HIV-induced inflammatory responses (22, 23). Until now, the relationship between HIF-1α and PRRSV infection has not been investigated.

In this study, we provide evidence that PRRSV infection elevates HIF-1α expression, which contributes to PRRSV replication. Detailed analyses demonstrated that PRRSV nonstructural protein 1β (nsp1β) stabilizes the expression of HIF-1α by preventing pVHL-mediated ubiquitination degradation. Furthermore, our data point to positive regulation of nsp1β stabilization by pVHL via K63-linked ubiquitination modification.

## RESULTS

### PRRSV infection promotes the expression of HIF-1α.

**FIG 1:**
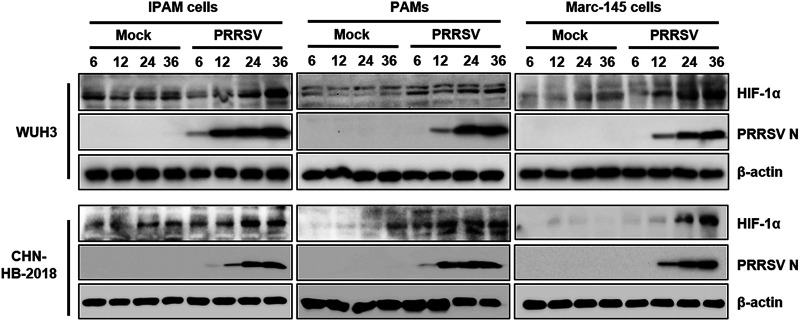
PRRSV infection regulates the expression of HIF-1α. Western blotting of HIF-1α and PRRSV N protein expression over time (hours) following strain WUH3 or CHN-HB-2018 infection (multiplicity of infection [MOI] of 0.5) or no infection of IPAM cells, PAMs, and Marc-145 cells.

Previous studies showed that certain viruses have evolved mechanisms to stabilize HIF-1α, such as SARS-CoV-2 and respiratory syncytial virus (RSV) (22, 24). To examine the response of HIF-1α to PRRSV infection, porcine alveolar macrophages (PAMs), IPAM cells (25), which are immortalized PAMs that are highly susceptible to PRRSV, and Marc-145 cells were infected with PRRSV strain WUH3, a highly pathogenic type 2 PRRSV. The kinetics of HIF-1α expression was tested at different time points in the hours following PRRSV infection. As shown by the results in Fig. 1, the expression levels of HIF-1α were increased during infection with PRRSV strain WUH3 in IPAM cells, PAMs, and Marc-145 cells compared with the level in uninfected cells. We also analyzed the expression kinetics of HIF-1α after infection with a strain NADC30-like PRRSV strain, CHN-HB-2018, and similar results were observed (Fig. 1). These results indicate that PRRSV infection significantly increased the expression of HIF-1α regardless of cell type.

### HIF-1α regulates PRRSV replication.

**FIG 2:**
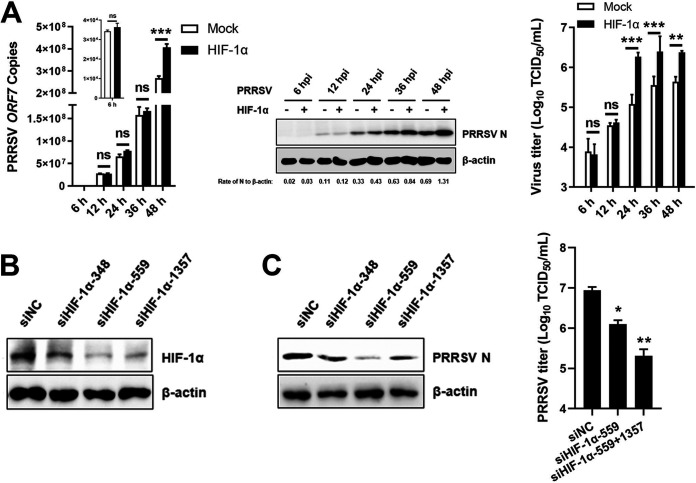
HIF-1α regulates PRRSV replication. (A) Viral yields in IPAM cells overexpressing HIF-1α at different time points after PRRSV infection (hours postinfection [hpi]) analyzed by qRT-PCR, Western blotting, and TCID_50_ assay. N protein expression detected by Western blotting was quantitatively estimated using ImageJ software (https://imagej.net/software/imagej/) and is presented as the density value relative to that of β-actin. (B) The knockdown efficiencies of three HIF-1α-specific siRNAs or control siRNAs (NC) against HIF-1α were analyzed by Western blotting at 36 h after transfection. (C) IPAM cells were transfected with HIF-1α-specific siRNA or control siRNA and then infected with PRRSV (MOI of 0.5) for 24 h. PRRSV N protein expression and viral titers were analyzed by Western blotting and TCID_50_ assay. *, *P* < 0.05; **, *P* < 0.01; ***, *P* < 0.001; ns, no significance (two-tailed Student’s *t* test for pairwise comparisons and one-way ANOVA for multiple comparisons). Data are mean values ± SEM from at least three biological replicates.

To determine whether HIF-1α is associated with PRRSV replication, IPAM cells were transiently transfected with an HIF-1α eukaryotic expression construct (pCAGGS-HIF-1α), followed by infection with PRRSV. As shown by the results in Fig. 2A, overexpression of HIF-1α dramatically increased viral nucleocapsid (N) protein expression levels and viral titers at 24, 36, and 48 h postinfection (hpi) but only slightly increased the viral RNA copy number at 48 hpi, suggesting that activation by HIF-1α mainly facilitates viral protein expression and viral titer. We next tested the effect of HIF-1α knockdown on PRRSV proliferation. Three small interfering RNAs (siRNAs) targeting HIF-1α were designed, and their knockdown efficiencies were measured by Western blot analysis. The siRNAs showed different knockdown efficiencies on HIF-1α expression, with siHIF-1α-559 and siHIF-1α-1357 displaying excellent efficiency (Fig. 2B). As expected, knockdown of endogenous HIF-1α by siHIF-1α led to an extreme reduction in PRRSV proliferation (Fig. 2C). Taken together, these results suggest that HIF-1α plays an important role in PRRSV infection.

### PRRSV nsp1β upregulates the expression of HIF-1α.

**FIG 3:**
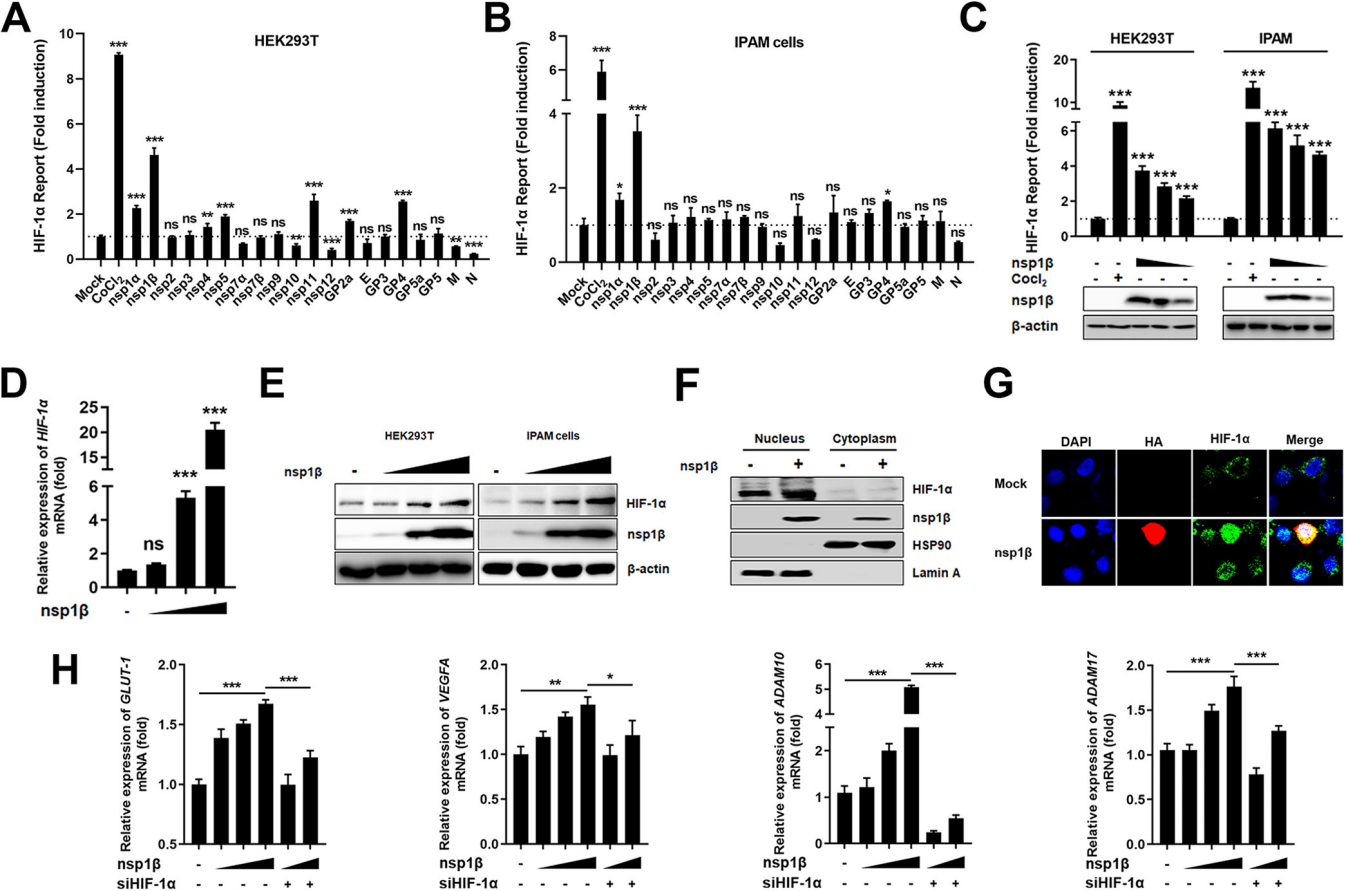
PRRSV nsp1β regulates HIF-1α expression. (A and B) Dual-luciferase assays of HEK293T cells (A) and IPAM cells (B) cotransfected with the indicated PRRSV protein overexpression plasmid (0.6 μg), the HIF-1α promoter reporter plasmid (0.2 μg), and the pRL-TK control plasmid (0.02 μg) for 36 h. (C) Dual-luciferase assays of HEK293T cells and IPAM cells cotransfected with PRRSV nsp1β (0.8, 0.4, or 0.2 μg), the HIF-1α reporter (0.2 μg), and pRL-TK plasmid (0.02 μg) for 36 h. (D and E) Expression of HIF-1α in HEK293T cells and IPAM cells transfected with PRRSV nsp1β (1, 2, or 4 μg) for 36 h, measured by qRT-PCR (D) and Western blotting (E). (F) Western blotting of HIF-1α and nsp1β in nuclear (lanes 1 and 2) and cytoplasmic (lanes 3 and 4) fractions of IPAM cells with or without overexpression of nsp1β. HSP90 and lamin A were used as nuclear and cytoplasmic markers, respectively. (G) IPAM cells were transfected with PRRSV nsp1β expression plasmid (1 μg) to examine the localization of HIF-1α by laser confocal assay. (H) Levels of *GLUT-1*, *VEGFA*, *ADAM10*, and *ADAM17* mRNA in IPAM cells transfected with PRRSV nsp1β (1, 2, or 4 μg) or PRRSV nsp1β followed by siRNA knockdown of HIF-1α. *, *P* < 0.05; **, *P* < 0.01; ***, *P* < 0.001; ns, no significance (two-tailed Student’s *t* test for pairwise comparisons and one-way ANOVA for multiple comparisons). Data are mean values ± SEM from at least three biological replicates.

To determine whether PRRSV-encoded proteins regulate HIF-1α expression, expression plasmids encoding individual structural and nonstructural proteins (nsp6 and nsp8 were not included because these two proteins are smaller, 16 amino acids and 45 amino acids, respectively) of PRRSV strain WUH3 were constructed, cotransfected with an HIF-1α promoter reporter plasmid (HIF-1α-pH800) into HEK293T cells and IPAM cells. As shown by the results in Fig. 3A to C, treatment with CoCl_2_, a chemical inducer of HIF-1α, significantly activated HIF-1α promoter activity (26). Overexpression of PRRSV nsp1β also increased HIF-1α promoter activity in both HEK293T cells and IPAM cells. Meanwhile, nsp1α and GP4 could also increase HIF-1α promoter activity, but to levels lower than the level induced by nsp1β (Fig. 3A and B). We then examined the expression of HIF-1α in HEK293T cells and IPAM cells after transfection with the nsp1β overexpression plasmid and found that overexpression of nsp1β increased HIF-1α mRNA and protein expression in a dose-dependent manner (Fig. 3D and E). Considering that HIF-1α must accumulate in the nucleus to act as a transcription factor (27), we tested whether nsp1β affected the subcellular localization of HIF-1α. As shown by the results in Fig. 3F and G, HIF-1α was recruited to the nucleus in IPAM cells and accumulated at a strikingly higher level in cells overexpressing nsp1β than in cells overexpressing empty vector, indicating that induction of HIF-1α by nsp1β may contribute to its activation.

We also examined the expression of HIF-1α downstream effector genes in IPAM cells overexpressing nsp1β and found that overexpression of nsp1β significantly upregulated the mRNA levels of *GLUT-1*, *VEGFA*, *ADAM10*, and *ADAM17* in a dose-dependent manner (Fig. 3H). Knockdown of HIF-1α markedly depressed the expression of all four HIF-1α downstream genes induced by nsp1β (Fig. 3H), indicating that nsp1β facilitated the expression of HIF-1α target genes through a strategy of increasing HIF-1α expression.

### Enzymatic activities of PRRSV nsp1β are essential for the stability of HIF-1α.

**FIG 4:**
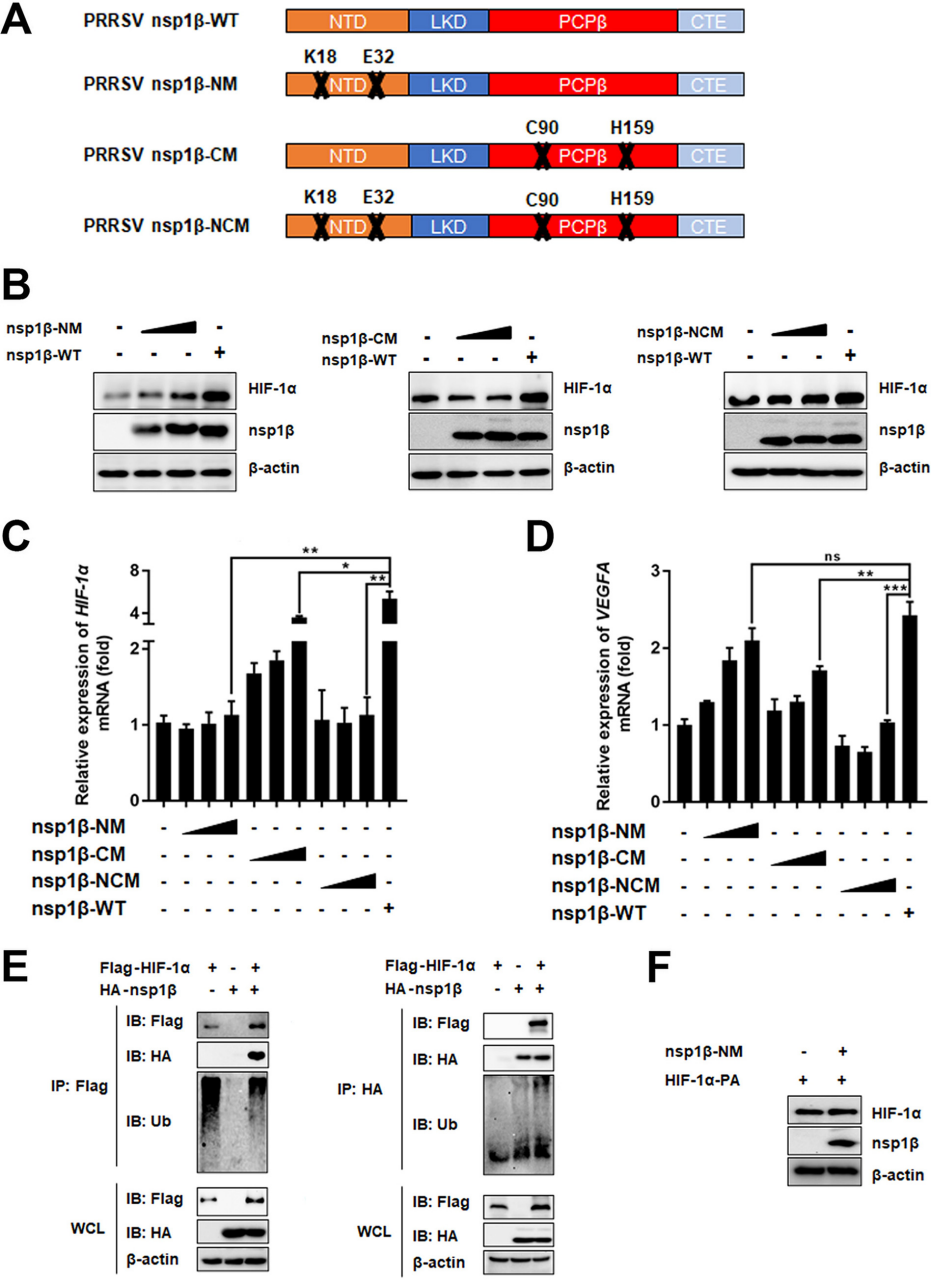
PRRSV nsp1β enzyme activities are involved in the stabilization of HIF-1α. (A) Schematic diagram of PRRSV nsp1β-WT (PDB ID 3MTV) and PRRSV nsp1β mutants. (B) Western blotting of IPAM cells transfected with plasmids expressing nsp1β-WT or mutant nsp1β-NM, nsp1β-CM, or nsp1β-NCM for 30 h to examine the expression of HIF-1α. (C and D) qRT-PCR of IPAM cells transfected with nsp1β-WT or mutant nsp1β-NM, nsp1β-CM, or nsp1β-NCM for 30 h to measure transcription levels of *HIF-1α* (C) and *VEGFA* (D). (E) Immunoblotting (IB) of HEK293T cells grown in 60-mm dishes and cotransfected with different combinations of HA-tagged nsp1β-WT (2 μg), FLAG-tagged HIF-1α (1.5 μg), and empty vector for 36 h. WCLs and IP complexes were analyzed using anti-FLAG, anti-HA, or anti-β-actin antibody. (F) Western blotting of WCLs of HEK293T cells grown in 60-mm dishes and cotransfected for 30 h with HA-tagged nsp1β-NM (2 μg) and FLAG-tagged HIF-1α-PA (2 μg) or empty vector. *, *P* < 0.05; **, *P* < 0.01; ***, *P* < 0.001; ns, no significance (two-tailed Student’s *t* test for pairwise comparisons and one-way ANOVA for multiple comparisons). Data are mean values ± SEM from at least three biological replicates.

The crystal structure of nsp1β reveals that its N-terminal domain (NTD) has intrinsic nuclease activity; however, certain features distinguish nsp1β from other characterized nucleases, including a C-terminal papain-like cysteine protease (PCPβ) domain, a linker domain that connects the NTD and PCPβ domain, and a C-terminal extension domain (PDB identifier [ID] 3MTV) (28). Previous studies of PRRSV nsp1β have demonstrated that the K18 and E32 amino acid residues within the NTD are critical for its nuclease activity, while C-terminal residues C90 and H159 are essential for its PCPβ activity (28). To determine whether PRRSV nsp1β increases HIF-1α expression in relation to its nuclease and/or PCPβ activities, we constructed three mutants for expression plasmids: nsp1β-NM, an N-terminal nuclease mutant inactivated by replacing K18 and E32 with Arg; nsp1β-CM, a C-terminal PCPβ mutant inactivated by replacing C90 and H159 with Arg; and nsp1β-NCM, a mutant with nuclease and PCPβ activity both inactivated by replacing K18, E32, C90, and H159 with Arg (Fig. 4A). The abilities of wild-type (WT) and enzyme-inactivated mutants of nsp1β to regulate HIF-1α expression were compared in IPAM cells. Although overexpression of nsp1β-NM was able to stabilize the HIF-1α protein in a dose-dependent manner, the ability to stabilize HIF-1α was significantly reduced compared to that of nsp1β-WT (Fig. 4B). In contrast, both nsp1β-CM and nsp1β-NCM lost the ability to stabilize HIF-1α compared with nsp1β-WT (Fig. 4B), indicating that both the nuclease protease and the PCPβ of nsp1β may be involved in the stabilization of HIF-1α.

Because nsp1β promoted HIF-1α expression at both the mRNA and protein levels (Fig. 3D and E), we further analyzed the potential roles of the nuclease and PCPβ activities of nsp1β in the transcription of *HIF-1α*. Transfection of IPAM cells with different doses of nsp1β-WT or mutant expression plasmids showed that the ability to promote HIF-1α transcription disappeared after transfection with nsp1β-NM or nsp1β-NCM (Fig. 4C), whereas nsp1β-CM still boosted HIF-1α transcription in a dose-dependent manner (Fig. 4C). These results suggested that the effect of nsp1β-induced HIF-1α transcription was dependent on the enzymatic activity of the N-terminal nuclease of nsp1β. We also analyzed the effect of nsp1β on the expression of *VEGFA*, a downstream effector gene of HIF-1α. Interestingly, nsp1β-NCM lost the ability to increase transcription of *VEGFA*, but both nsp1β-NM and nsp1β-CM still promoted *VEGFA* transcription in a dose-dependent manner, albeit at slightly reduced levels compared with nsp1β-WT (Fig. 4D). Because nsp1β-NM lost the ability to induce HIF-1α transcription but still increased the expression of an HIF-1α target gene, it is possible that the C-terminal PCPβ activity of nsp1β regulated HIF-1α expression at the protein level.

Because HIF-1α activity is suppressed by factor inhibiting HIF-1 (FIH-1) through hydroxylation of HIF-1α N803, which blocks its binding to coactivators p300/CBP (21), we investigated whether PRRSV nsp1β regulated the expression of FIH-1. Overexpression of nsp1β did not affect the mRNA or protein expression of FIH-1 in IPAM cells (Fig. S1A and B in the supplemental material), demonstrating that nsp1β-mediated regulation of HIF-1α expression may not operate through an FIH-1 strategy. Furthermore, HIF-1α’s function is strictly regulated by pVHL through ubiquitination degradation. Because the C-terminal PCPβ activity of nsp1β stabilized the expression of HIF-1α, we proposed that it might be involved in removing the polyubiquitin chain of HIF-1α. To this end, we first determined whether nsp1β possessed deubiquitylation (DUB) enzyme activity. HEK293T cells were cotransfected with a ubiquitin (Ub) expression plasmid and nsp1β-WT or its mutants. A construct that expresses PRRSV nsp2, which was previously demonstrated to have DUB activity (29), was used as a positive control. As shown by the results in Fig. S2A and B, while nsp1β-WT and nsp1β-NM were able to cleave polyubiquitinated chains in a dose-dependent manner, this ability had almost disappeared in nsp1β-CM and nsp1β-NCM. These results confirmed that PRRSV nsp1β was a potent DUB enzyme that removed Ub conjugates from cellular substrates via its C-terminal PCPβ activity. Next, we tested whether the C-terminal DUB ability of nsp1β was involved in the degradation of polyubiquitin chains bound to HIF-1α. To explore the regulatory role of nsp1β on HIF-1α protein expression separately from its effect on the endogenous *HIF-1α* promoter, we used a strategy of FLAG-tagged HIF-1α overexpression. FLAG-tagged HIF-1α and hemagglutinin (HA)-tagged nsp1β expression plasmids were cotransfected into HEK293T cells for coimmunoprecipitation (IP) experiments. We found that overexpression of nsp1β significantly inhibited the ubiquitination of HIF-1α and increased the expression of HIF-1α (Fig. 4E). In contrast, the nsp1β-NM mutant could not increase the expression of HIF-1α-PA, an HIF-1α mutant that escapes pVHL-mediated ubiquitylation degradation (Fig. 4F) (30), suggesting that the DUB activity of nsp1β played a vital role in the stability of HIF-1α. Taken together, these results suggest that nsp1β stabilized HIF-1α expression by promoting the transcription of HIF-1α through its N-terminal nuclease domain and by cleaving the polyubiquitin chain bound to HIF-1α through the DUB capacity of the nsp1β C-terminal PCPβ.

### nsp1β, HIF-1α, and pVHL form a ternary complex.

**FIG 5:**
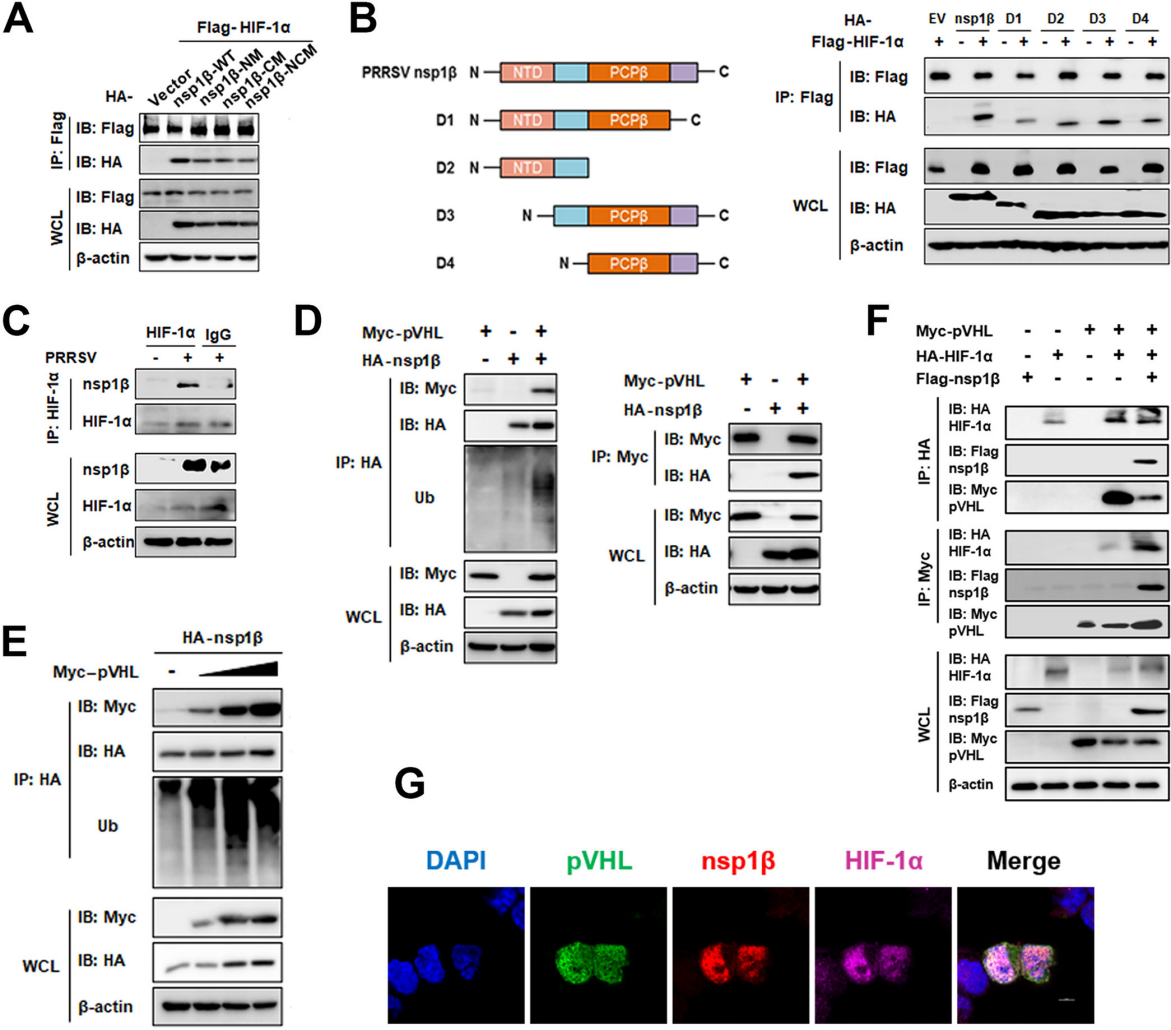
nsp1β, HIF-1α, and pVHL form a ternary complex. (A) IB of HEK293T cells transfected with expression plasmids encoding HA-tagged nsp1β-WT or mutant nsp1β-NM, nsp1β-CM, or nsp1β-NCM and FLAG-tagged HIF-1α for 30 h. WCLs and IP complexes were analyzed using anti-FLAG, anti-HA, or anti-β-actin antibody. (B) Left, schematic illustration of full-length and truncated constructs (D1 to D4) of PRRSV strain WUH3 nsp1β. Right, plasmids expressing the nsp1β-WT or truncated mutants and HIF-1α were cotransfected into HEK293T cells for 30 h. WCLs were subjected to IP with anti-FLAG antibody, and WCLs and IP complexes were analyzed by IB using anti-FLAG, anti-HA, or anti-β-actin antibody. (C) PAMs infected with PRRSV were lysed at 30 hpi and subjected to IP with anti-HIF-1α antibody. WCLs and IP complexes were analyzed by Western blotting. (D and E) HEK293T cells cotransfected with expression plasmids encoding HA-tagged nsp1β and Myc-tagged pVHL (D) or different doses of Myc-tagged pVHL (E) were subjected to IP with anti-HA or anti-Myc antibody. WCLs and IP complexes were analyzed by Western blotting. (F) HEK293T cells cotransfected with HA-tagged HIF-1α, FLAG-tagged nsp1β, and Myc-tagged pVHL were subjected to IP with anti-HA or anti-Myc antibody 30 h after transfection. WCL and IP complexes were analyzed by Western blotting. (G) HEK293T cells were cotransfected with FLAG-nsp1β, HA-HIF-1α, and Myc-pVHL expression plasmids for 24 h. The cells were fixed and subjected to immunofluorescence assay.

To investigate whether the interaction between HIF-1α and nsp1β was dependent on the enzymatic activities of nsp1β, the interactions between HIF-1α and nsp1β mutants were examined using co-IP. Like the nsp1β-WT, the nsp1β-NM, nsp1β-CM, and nsp1β-NCM mutants were able to interact with HIF-1α (Fig. 5A), indicating that these interactions were independent of nsp1β enzymatic activities. Next, to identify which domain(s) of nsp1β was responsible for the nsp1β–HIF-1α interaction, we generated four truncation mutants of nsp1β (nsp1β-D1, -D2, -D3, and -D4) (Fig. 5B). Co-IP experiments on HEK293T whole-cell lysates (WCLs) confirmed that all four truncation mutants were able to interact with HIF-1α (Fig. 5B). To determine the physiological relevance of these findings in the context of viral infection, anti-HIF-1α antibody was used for IP of PRRSV nsp1β from the WCLs of PRRSV-infected cells. As shown by the results in Fig. 5C, PRRSV nsp1β was coimmunoprecipitated with HIF-1α.

We found it interesting that not only did nsp1β interact with HIF-1α, but the polyubiquitin chain bound to nsp1β was also increased upon cotransfection of nsp1β and HIF-1α (Fig. 5E). Since our data from previous Ub analysis of PRRSV-infected PAMs showed that nsp1β might be ubiquitinated (31), we confirmed here that nsp1β was stabilized by cotransfection with Ub and nsp1β in HEK293T cells (Fig. S3A). Knowing that HIF-1α does not possess E3 Ub ligase activity, we speculated that pVHL, an E3 Ub ligase enzyme involved in HIF-1α degradation (13), might be involved in the complex of nsp1β and HIF-1α to exercise the E3 ligase function. To test whether nsp1β interacted with pVHL, Myc-tagged pVHL and HA-tagged nsp1β expression plasmids were cotransfected into HEK293T cells and assayed for IP with antibodies against HA and Myc. As shown by the results in Fig. 5D, pVHL interacted with nsp1β and induced polyubiquitination of nsp1β in a dose-dependent manner (Fig. 5E). These results suggested that pVHL enhanced the stabilization of nsp1β by increasing the ubiquitination of nsp1β, which may be an alternative substrate for pVHL.

Discovering that, while the interaction between nsp1β and HIF-1α promoted the expression of HIF-1α, the interaction between nsp1β and pVHL boosted the stabilization of nsp1β led us to speculate that the binding of HIF-1α to pVHL and its binding to nsp1β might be mutually exclusive. To address this possibility, HEK293T cells were cotransfected with plasmids encoding Myc-pVHL, HA-HIF-1α, and FLAG-nsp1β. Myc-pVHL and HA-HIF-1α were immunoprecipitated and then subjected to immunoblotting (IB) analysis. As shown by the results in Fig. 5F, pVHL, HIF-1α, and nsp1β formed a ternary complex. Furthermore, the amount of Myc-pVHL bound to HA-HIF-1α decreased sharply in the presence of nsp1β, suggesting that nsp1β might impair the recruitment of HIF-1α by pVHL. An immunofluorescence assay also showed that there exists colocalization among nsp1β, HIF-1α, and pVHL (Fig. 5G). Altogether, these results revealed a possible role for this ternary complex, wherein nsp1β impairs the interaction between HIF-1α and pVHL, which may hinder the ubiquitination degradation of HIF-1α by pVHL, but also, nsp1β recruits pVHL, using its E3 ligase activity to stabilize nsp1β expression through ubiquitination modification.

### pVHL mediates K63-linked ubiquitination of PRRSV nsp1β at Lys18, -36, and -189.

**FIG 6:**
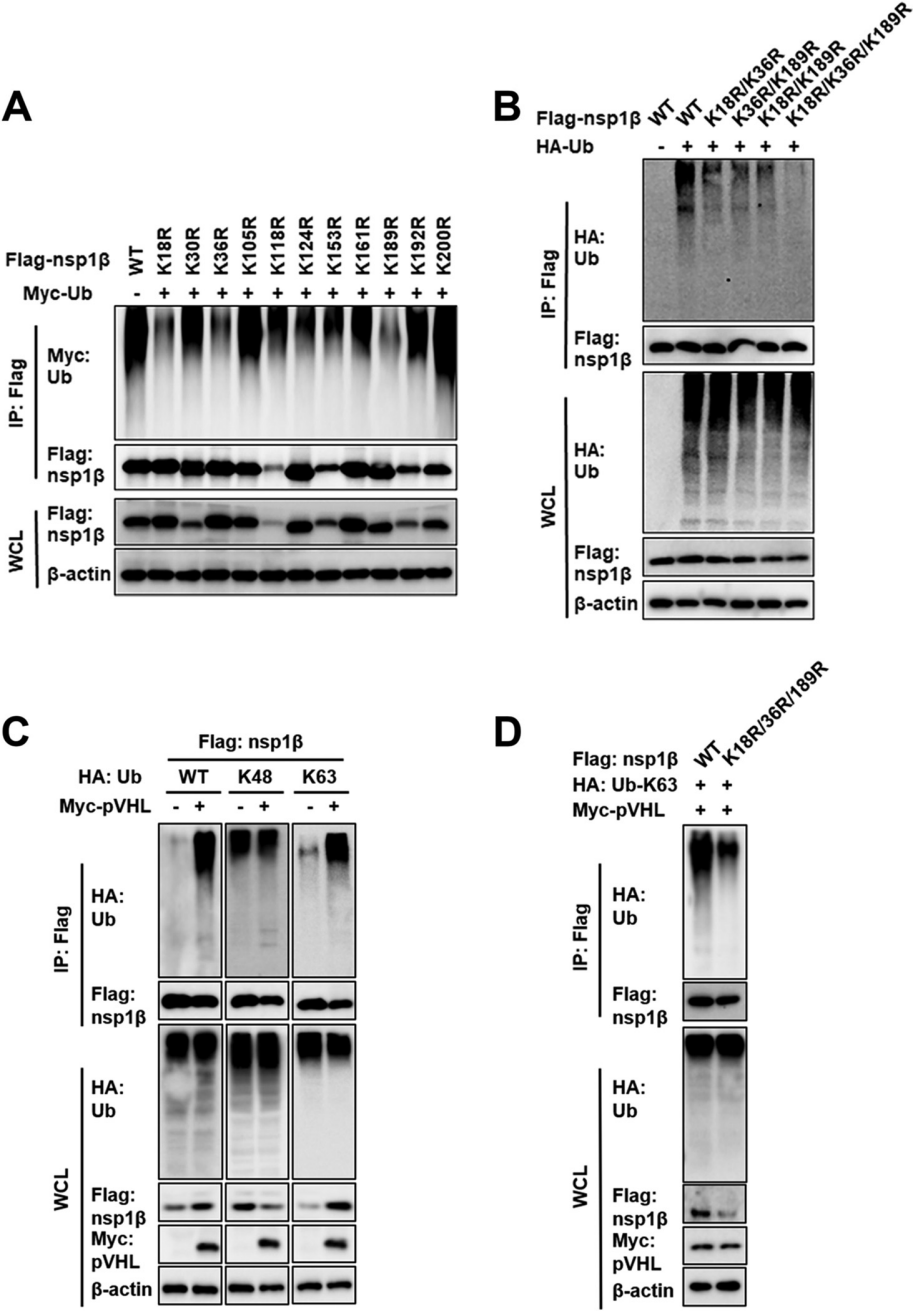
pVHL positively regulates nsp1β by direct conjugation of K63-linked Ub chains at Lys18, -36 and -189. (A and B) HEK293T cells cotransfected with expression plasmids carrying FLAG-tagged nsp1β-WT or mutant (nsp1β K18R/K36R, K36R/K189R, K18R/K189R, or K18R/K36R/K189R) and Myc-tagged Ub (A) or HA-tagged Ub (B) for 30 h were subjected to IP with anti-FLAG antibody. WCLs and IP complexes were analyzed by Western blotting. (C) HEK293T cells cotransfected with FLAG-nsp1β, Myc-pVHL, and HA-Ub (WT, K48, or K63) for 30 h were subjected to IP using anti-FLAG antibody and then analyzed by IB with an anti-HA or anti-FLAG antibody. Levels of transfected proteins were analyzed by IB with an anti-HA, anti-Myc, or anti-FLAG antibody. (D) HEK293T cells cotransfected with Myc-pVHL, FLAG-nsp1β-WT, or mutant (Lys18, -36, or -189 to Arg), and HA-Ub WT or mutant (K63) for 30 h were subjected to IP with anti-FLAG antibody. WCLs and IP complexes were analyzed by Western blotting.

To explore which lysine residues of nsp1β might be modified by ubiquitination, we analyzed the nsp1β amino acid sequence of PRRSV strain WUH3 and identified 11 lysine residues that were conserved in different PRRSV isolates. Next, we constructed 11 nsp1β mutants bearing single Lys-to-Arg substitutions at each potential ubiquitination site. Plasmids expressing Myc-tagged Ub and FLAG-tagged WT or mutant nsp1β constructs were cotransfected into HEK293T cells, followed by IP of nsp1β with anti-FLAG antibody. We found that the amounts of Ub bound on three of the nsp1β mutants, those bearing mutations of Lys to Arg at position 18 (K18R), K36R, and K189R, were significantly reduced compared with the amount bound on nsp1β-WT, while all other mutants retained the ability to conjugate Ub in co-IP experiments (Fig. 6A). We then generated the following combination mutations: nsp1β K18R/K36R, K36R/K189R, K18R/K189R, and K18R/K36R/K189R. As shown by the results in Fig. 6B, the ubiquitination of the nsp1β K18R/K36R/K189R triple mutant was almost completely abolished compared with that of nsp1β-WT and the other nsp1β mutants (K18R/K36R, K36R/K189R, and K18R/K189R) (Fig. 6B), indicating Lys18, -36 and -189 as the major sites of ubiquitination in nsp1β.

To identify the specific Ub chain forms of pVHL-mediated ubiquitination of nsp1β, we first constructed HA-tagged Ub mutant expression plasmids in which all lysine residues except K48 or K63 were mutated to arginine (HA-K48-Ub or HA-K63-Ub). HEK293T cells were transfected with HA-WT-Ub, HA-K48-Ub, or HA-K63-Ub together with FLAG-nsp1β and Myc-pVHL. As shown by the results in Fig. 6C, pVHL substantially increased nsp1β polyubiquitination in cells transfected with plasmids encoding the WT Ub and the K63 mutant, but not the K48 mutant, indicating that pVHL mediates K63-linked polyubiquitination of nsp1β. Furthermore, cotransfection of FLAG-nsp1β-WT or FLAG-nsp1β K18R/K36R/K189R with HA-K63-Ub and Myc-pVHL in HEK293T cells revealed a significant reduction in polyubiquitin chains bound to the nsp1β K18R/K36R/K189R mutant compared with the amount bound to nsp1β-WT (Fig. 6D). Taken together, these results reveal that pVHL is an E3 ligase that positively regulates nsp1β expression by binding the K63-linked Ub chain to the Lys18, -36, and -189 sites of nsp1β.

### Accumulated HIF-1α upregulates PRRSV-induced inflammatory responses.

**FIG 7:**
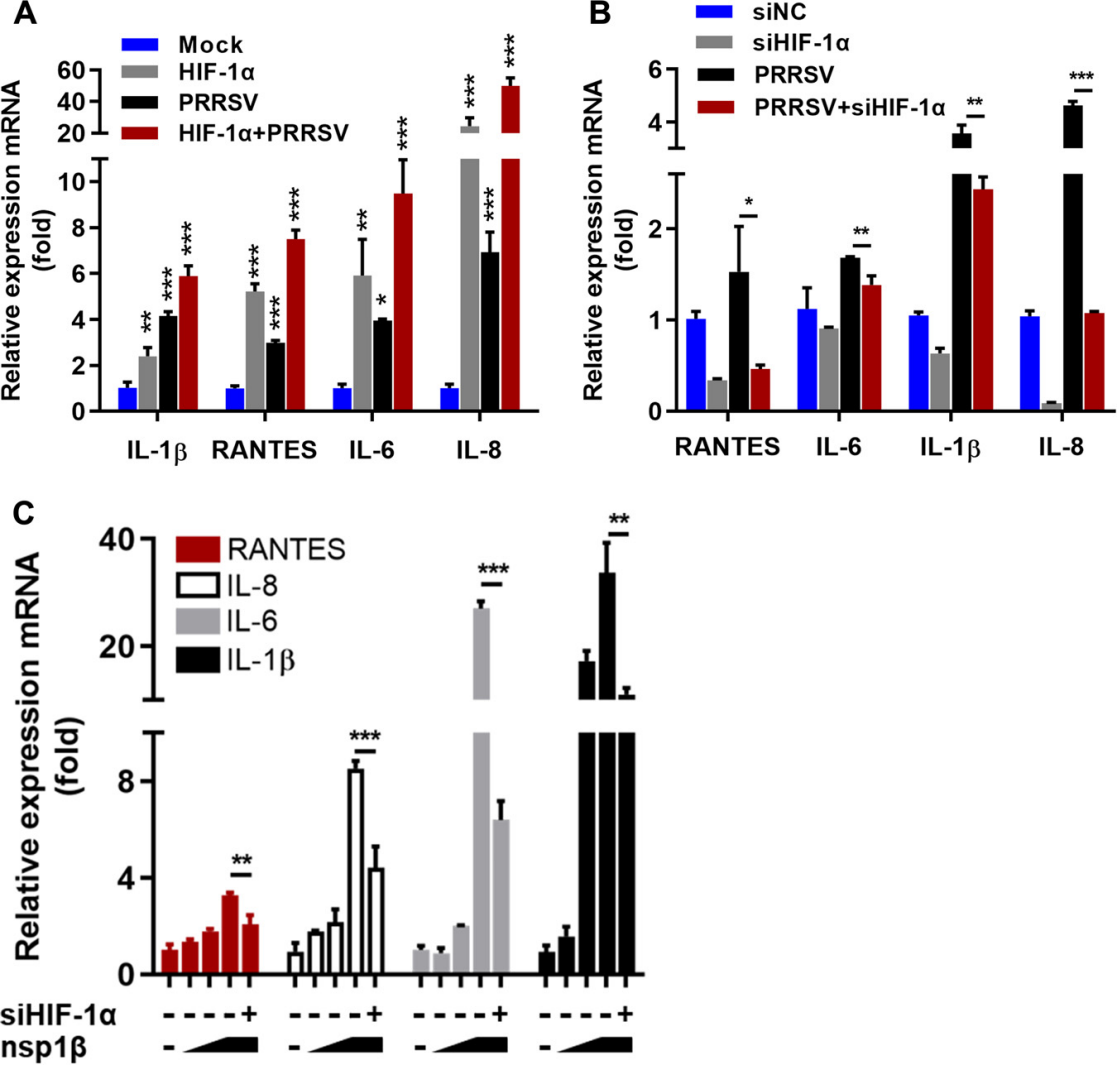
HIF-1α is involved in the PRRSV-induced inflammatory response. (A) IPAM cells transfected with HIF-1α or infected with PRRSV and then transfected with HIF-1α were assessed by qRT-PCR of relative mRNA expression of inflammatory factor genes *RANTES*, *IL-6*, *IL-8*, and *IL-1β*. (B) qRT-PCR of relative mRNA expression of inflammatory factor genes in IPAM cells with knockdown of HIF-1α by siRNA, with or without PRRSV infection. (C) IPAM cells transfected with PRRSV nsp1β (1, 2, or 4 μg) or transfected with PRRSV nsp1β followed by knockdown of HIF-1α were assessed by qRT-PCR of relative mRNA expression of inflammatory factor genes. *, *P* < 0.05; **, *P* < 0.01; ***, *P* < 0.001 (two-tailed Student’s *t* test for pairwise comparisons and one-way ANOVA for multiple comparisons). Data are mean values ± SEM from at least three biological replicates.

Previous studies have shown that HIF-1α is an important factor in the regulation of inflammation (32) and that interstitial pneumonia is a typical pathological characteristic of PRRSV-infected pigs. Having shown that PRRSV induced enhanced HIF-1α expression (Fig. 1A), we investigated whether HIF-1α affected PRRSV-induced inflammatory responses. As shown by the results in Fig. 7A, overexpression of HIF-1α significantly enhanced the expression of *RANTES*, *IL-6*, *IL-8*, and *IL-1β* (Fig. 7A). Overexpression of HIF-1α in response to PRRSV infection further increased the PRRSV-induced inflammatory responses (Fig. 7A). In contrast, knockdown of HIF-1α impaired inflammatory cytokine expression and PRRSV-induced inflammatory cytokine expression (Fig. 7B). These results suggest that PRRSV promotes the expression of inflammatory cytokines by hijacking HIF-1α.

Previous studies suggested that PRRSV nsp1β was involved in the regulation of inflammatory responses (33, 34). Because our data showed that PRRSV nsp1β upregulated HIF-1α expression, we speculated that PRRSV nsp1β might also positively regulate the expression of inflammatory cytokine genes. As expected, overexpression of nsp1β remarkably boosted the mRNA expression of *RANTES*, *IL-6*, *IL-8*, and *IL-1β* in a dose-dependent manner (Fig. 7C). Furthermore, knockdown of HIF-1α decreased the mRNA levels of *RANTES*, *IL-6*, *IL-8*, and *IL-1β* induced by nsp1β (Fig. 7C), suggesting that nsp1β facilitates the expression of inflammatory cytokines by increasing HIF-1α expression. These results suggest that PRRSV nsp1β might be a proinflammatory protein that facilitates the expression of inflammatory cytokines through HIF-1α signaling.

## DISCUSSION

It is an important strategy for virus survival and replication that viruses hijack host factors and then exploit their functions (21). A better understanding of virus-host interactions may provide new potential approaches for the treatment of viral infection. In this study, our results uncovered HIF-1α as a virus-dependent host factor important for optimal infection of macrophages by PRRSV, suggesting a potential new anti-PRRSV target.

PRRSV infection causes lung parenchymal injury characterized by alveolar wall thickening, vascular hyperpermeability, and inflammatory cell infiltration, leading to the development of hypoxemia (35). Under hypoxia, PHD enzymes are inhibited, leading to HIF-1α stabilization; once stabilized, HIF-1α dimerizes with HIF-1β to bind to hypoxia-responsive elements, thus inducing the expression of target genes that are useful under hypoxic conditions (21). These target genes are involved in the production of inflammatory cytokines (36). Indeed, we found that PRRSV infection stabilized HIF-1α, which in turn promoted cellular inflammatory responses, highlighting the important role of HIF-1α in PRRSV infection.

Notably, there is evidence that PRRSV infection upregulates the expression of a variety of pro-inflammatory factors that play important roles in viral infection and pathogenesis (10). The E and N proteins of PRRSV, in particular, are capable of markedly upregulating the expression of inflammatory cytokines, including RANTES, IL-8, IL-6, and IL-1β, leading to a cytokine storm (37–42). In the present study, overexpression of nsp1β significantly promoted the mRNA expression of inflammatory genes *IL-6*, *IL-8*, *RANTES*, and *IL-1β* in a dose-dependent manner in IPAM cells. Moreover, a recent study has also shown that PRRSV nsp1β is a proinflammatory viral protein (33).

The genome of PRRSV encodes at least 10 open reading frames (ORFs) (43). ORF1a and ORF1ab encode two large polyproteins predicted to be cleaved into 14 mature nonstructural proteins (nsp’s) (44). Among these nsp’s, nsp1 can be autocleaved into nsp1α and nsp1β (28). To date, most studies on PRRSV nsp1β have focused on the field of inhibition of interferon (IFN) production by the N-terminal nuclease activity of nsp1β (45). Because the C-terminal PCPβ structural domain of nsp1β did not reportedly affect IFN production (46, 47), the role played by PCPβ during the course of viral infection was theretofore unresolved. In the present study, we revealed that the PRRSV nsp1β PCPβ structural domain possesses DUB activity. Based on this DUB activity, we speculate that nsp1β may have the ability to stabilize certain virus-dependent host factors that are degraded by ubiquitination, thereby facilitating self-proliferation. For example, nsp1β stabilized HIF-1α through its DUB activity, and that stabilization of HIF-1α facilitated PRRSV replication, which may explain the classification of nsp1β as a PRRSV virulence factor. To assess their roles in the PRRSV life cycle, substitutions and deletions of the enzymatically active sites of the NTD and PCPβ were introduced into infectious cDNA clones of PRRSV. Unfortunately, mutants in which the NTD and/or PCPβ active sites were blocked proved to be nonviable and produced no detectable sign of viral RNA synthesis, indicating that the correct processing of the enzymatically active sites of the NTD and PCPβ was essential for PRRSV genome replication (46, 48).

Infection with some respiratory viruses, including PRRSV and SARS-CoV-2, results in a severe respiratory inflammatory response, which in turn leads to a cytokine storm (22, 49). A fast, well-controlled innate immune response is the first line of defense against respiratory viral infections, but sustained and uncontrolled inflammatory responses lead to tissue damage and a series of secondary infections (50). Therefore, a better understanding of the mechanism controlling immune-mediated protection against immune-induced tissue damage is urgently required to develop effective host-targeted therapeutics against severe viral pneumonia. PRRSV, a pathogen that induces a strong immune response, might be a good model for studying the reprogramming of the immune system.

In conclusion, these data reveal a novel mechanism by which PRRSV infection increases HIF-1α levels. Mechanistically, viral nsp1β promotes the stability of HIF-1α by its N-terminal nuclease activity and C-terminal DUB enzyme activity. Stabilized HIF-1α plays important roles in PRRSV growth and inflammatory responses. These findings indicate HIF-1α as a potential therapeutic target for PRRSV and suggest a new strategy for improving PRRSV culture systems.

## MATERIALS AND METHODS

### Cell culture and viruses.

PAMs, the target cells of PRRSV *in vivo*, were preserved in our laboratory as described previously (51). IPAM cells were kindly provided by Xue-Hui Cai (25). Both PAMs and IPAM cells were cultured in RPMI 1640 medium (Invitrogen, USA) supplemented with 10% heated-inactivated fetal bovine serum (FBS) at 37°C in a humidified 5% CO_2_ incubator. Marc-145 cells and HEK293T cells were cultured and maintained in Dulbecco’s modified Eagle’s medium (Invitrogen) supplemented with 10% FBS at 37°C in a humidified 5% CO_2_ incubator. PRRSV strain WUH3 is a highly pathogenic type 2 PRRSV (North America) isolated from the brains of pigs suffering from “high fever syndrome” in China in late 2006 (GenBank accession number HM853673). PRRSV strain CHN-HB-2018 (GenBank accession number MZ043753), an NADC30-like PRRSV, was isolated in China in 2018. The virus was amplified and titers determined in Marc-145 cells, a monkey kidney cell line highly permissive for PRRSV infection.

### Plasmid construction and siRNA synthesis and interference.

Expression plasmids for FLAG-tagged, HA-tagged, and Myc-tagged HIF-1α and Myc-tagged pVHL were constructed in the pCAGGS vector (52). The construction of HA-tagged expression plasmids (pCAGGS-HA) encoding the PRRSV nonstructural and structural proteins used in this study and the HA tag IB used to confirm the expression of each viral protein was described elsewhere (52, 53). Plasmids encoding truncated nsp1β mutants were described by Wang et al. (45). Construction of the luciferase reporter driven by a 0.8-kb fragment containing the HIF-1α 5′ untranslated region (287 bp) and pH800 promoter region (+1 to −541) was described previously (26). The following mutant nsp1β expression plasmids were newly constructed: pCAGGS-HA-nsp1β K18A/E32A (nsp1β-NM), pCAGGS-HA-nsp1β C90A/H159A (nsp1β-CM), pCAGGS-HA-nsp1β K18A/E32A/C90A/H159A (nsp1β-NCM), pCAGGS-FLAG-nsp1β-K18R/K36R, pCAGGS-FLAG-nsp1β-K36R/K189R, pCAGGS-FLAG-nsp1β-K18R/K189R, and pCAGGS-FLAG-nsp1β-K18R/K36R/K189R. The HIF-1α mutant plasmid containing two alanine substitutions in place of proline at positions P402 and P564 (HIF-1α-PA, which escapes pVHL-mediated degradation), was previously described (30). All constructs were confirmed by DNA sequencing. The siRNAs targeting pig HIF-1α and the negative-control siRNA were synthesized by GenePharma (Shanghai, China). The siRNA sequences used were as follows (5′ to 3′): HIF-1α-348-GCGUGUGAGGAAACUUCUATT; HIF-1α-559-CCGUGCGACCAUGAGGAAATT; HIF-1α-1357-GCUGGAGACACAAUCAUAUTT, and negative-control siRNA UUCUUCGAACGUGUCACGUTT. Transient transfections of expression plasmids and siRNAs were performed using Lipofectamine 3000 (Invitrogen) according to the manufacturer’s instructions.

### Antibodies and reagents.

Monoclonal antibody (MAb) against PRRSV nucleocapsid (N) was prepared in our laboratory as described previously (51, 54). Mouse or rabbit MAbs against FLAG, HA, and β-actin were purchased from MBL and used for IP and Western blot assay. Mouse MAb against HIF-1α (ab16066), which was used for IP and Western blot assay, and rabbit anti-heat shock protein 90 (HSP90) (ab59459), anti-lamin A (ab26300), and anti-FIH-1 (ab233141) antibodies, which were used for Western blot assay, were purchased from Abcam. Rabbit polyclonal antibody against Ub (#3936), which was used for Western blot assay, was purchased from Cell Signaling Technology. CoCl_2_ was purchased from Sigma-Aldrich (St. Louis, MO, USA).

### RNA extraction and qRT-PCR.

Total RNA was extracted from cultured cells using TRIzol reagent (Invitrogen) and was reverse transcribed into cDNA using reverse transcriptase (Vazyme). Real-time reverse transcriptase quantitative PCR (qRT-PCR) experiments were performed in triplicate. Relative mRNA expression levels were normalized to the expression of GAPDH (glyceraldehyde-3-phosphate dehydrogenase). Absolute mRNA levels were calculated using standard curves. All qRT-PCR experiments were performed using Power SYBR green PCR master mix (Applied Biosystems) and an ABI 7500 real-time PCR system (Applied Biosystems). The specific primer sequences used in this study are listed in Table 1.

**TABLE 1:** qRT-PCR primers used in the study

Primer^*a*^	Sequence (5′→3′)
PRRSV-ORF7-F	GCAATTGTGTCTGTCGTC
PRRSV-ORF7-R	CTTATCCTCCCTGAATCTGAC
GAPDH-F	ACATGGCCTCCAAGGAGTAAGA
GAPDH-R	GATCGAGTTGGGGCTGTGACT
IL-1β-F	AGTCTGCCCTGTACCCCAACT
IL-1β-R	ATCTTGGCGGCCTTTGGAGTT
IL-6-F	CTGCTTCTGGTGATGGCTACTG
IL-6-R	GGCATCACCTTTGGCATCTT
IL-8-F	AGTTTTCCTGCTTTCTGCAGCT
IL-8-R	TGGCATCGAAGTTCTGCACT
RANTES-F	ACACCCTGCTGTTTTTCCTACCT
RANTES-R	AGACGACTGCTGCCATGGA
HIF-1α-F	CATCAGTTGCCACTTCCCCAT
HIF-1α-R	CAAAACCATCCAAGGCTTTCA
GLUT-1-F	CACTGTCGTGTCGCTGTTC
GLUT-1-R	ATGCTCAGGTAGGACATCCAG
VEGFA-F	CGACGAAGGTCTGGAGTGT
VEGFA-R	TGTGCTGTAGGAAGCTCATCT
ADAM10-F	GACTGTAATAGGCATACGCAAGT
ADAM10-R	GTTAATGGCTCCATCTTCTTCATAC
ADAM17-F	GCGTGGATAAGAAGCTGGATAA
ADAM17-R	GATGCGAACGGATGCTGAAT
FIH-1-F	TGGTGGCATCACATAGAGTCAT
FIH-1-R	CCAAGCATCTTCTCAATGTTCCT

a F, forward; R, reverse.

### Western blotting and co-IP assay.

Samples were lysed with radioimmunoprecipitation assay (RIPA) lysis buffer containing 1 mM phenylmethylsulfonyl fluoride protease inhibitor (Beyotime) for 20 min and then separated by centrifugation for 10 min at 4°C; supernatants were denatured in 5× sample loading buffer for 10 min at 98°C. For IP assays, supernatants were mixed with the respective primary antibodies in the presence of protein A+G-agarose (Beyotime) at 4°C overnight, followed by Western blotting.

### Indirect immunofluorescence assay.

Cells were seeded on circular glass coverslips in 24-well plates and grown to 60% to 70% confluence. At the indicated time points after treatment, the cells were incubated in 4% paraformaldehyde for 15 min and immediately permeabilized with precooled methanol for 10 min. The cells were blocked with 5% bovine serum albumin in phosphate-buffered saline for 1 h and then incubated with the indicated antibodies for 1 h. The cells were treated with secondary antibodies for 1 h and then with DAPI (4′,6-diamidino-2-phenylindole; Beyotime, Nantong, China) in phosphate-buffered saline (1/400 dilution) for 15 min. The fluorescence images were acquired with an Olympus FV10 laser scanning confocal microscope (Olympus, Tokyo, Japan).

### Deubiquitylation (DUB) activity assay.

HEK293T cells cultured in 60-mm dishes were cotransfected with 2 μg of Ub expression plasmid plus the indicated amounts of plasmids encoding PRRSV nsp1β-WT or mutants for 30 h. Cells were harvested and then analyzed for the expression of Ub-conjugated proteins by Western blotting.

### Luciferase reporter assay.

HEK293T cells and IPAM cells grown in 48-well plates were transiently cotransfected with 0.2 μg of HIF-1α-pH800 promoter reporter plasmid (26) and 0.02 μg of phRL-TK luciferase control plasmid, together with 0.6 μg of each plasmid encoding a structural or nonstructural protein derived from PRRSV strain WUH3. Cells were then lysed, and firefly luciferase and *Renilla* luciferase activities were determined using the dual-luciferase reporter assay system (Promega) according to the manufacturer’s protocol. Data are presented as relative firefly luciferase activity normalized to *Renilla* luciferase activity and are representative of three independent experiments.

### TCID_50_ assay.

PRRSV titers are expressed as 50% tissue culture infectious doses (TCID_50_)/mL using the Reed-Muench method. Briefly, Marc-145 cells were seeded in 96-well plates and then infected with serial 10-fold dilutions of PRRSV samples in eight replicates. Plates were incubated for 120 h to 144 h before virus titers were calculated.

### Data analysis.

Data were obtained from three independent, reproducible experiments. Results are presented as the mean values ± standard errors of the means (SEM) from three independent experiments. Statistical significance was determined using the two-tailed Student’s *t* test for pairwise comparisons and one-way analysis of variance (ANOVA) for multiple comparisons. A *P* value of less than 0.05 was considered significant.
